# Prognostic Modeling and Prevention of Diabetes Using Machine Learning Technique

**DOI:** 10.1038/s41598-019-49563-6

**Published:** 2019-09-24

**Authors:** Sajida Perveen, Muhammad Shahbaz, Karim Keshavjee, Aziz Guergachi

**Affiliations:** 1 Department of Computer Science & Engineering, University of Engineering & Technology, Lahore, Pakistan; 2 Research Lab for Advanced System Modelling, Ryerson University, Toronto, Ontario Canada; 3 Institute for Health Policy, Management and Evaluation, University of Toronto, Toronto, Ontario Canada; 4 Ted Rogers School of Information Technology Management, Ryerson University, Toronto, Ontario Canada; 5 Department of Mathematics & Statistics, York University, Toronto, Ontario Canada

**Keywords:** Preventive medicine, Population screening

## Abstract

Stratifying individuals at risk for developing diabetes could enable targeted delivery of interventional programs to those at highest risk, while avoiding the effort and costs of prevention and treatment in those at low risk. The objective of this study was to explore the potential role of a Hidden Markov Model (HMM), a machine learning technique, in validating the performance of the Framingham Diabetes Risk Scoring Model (FDRSM), a well-respected prognostic model. Can HMM predict 8-year risk of developing diabetes in an individual effectively? To our knowledge, no study has attempted use of HMM to validate the performance of FDRSM. We used Electronic Medical Record (EMR) data, of 172,168 primary care patients to derive the 8-year risk of developing diabetes in an individual using HMM. The Area Under Receiver Operating Characteristic Curve (AROC) in our study sample of 911 individuals for whom all risk factors and follow up data were available is 86.9% compared to AROCs of 78.6% and 85% reported in a previously conducted validation study of FDRSM in the same Canadian population and the Framingham study respectively. These results demonstrate that the discrimination capability of our proposed HMM is superior to the validation study conducted using the FDRSM in a Canadian population and in the Framingham population. We conclude that HMM is capable of identifying patients at increased risk of developing diabetes within the next 8-years.

## Introduction

Diabetes mellitus is a chronic and lifelong metabolic disorder^1^ that occurs either when the pancreas does not secret enough insulin, due to destruction of pancreatic beta cells by T cells through an autoimmune mechanism, precipitating insulin-dependent/type 1 diabetes^2^, or when the body’s cells do not respond to insulin as effectively as they once did and unable to properly utilize the energy produced from the food, resulting in elevated levels of glucose circulating in the blood, otherwise known as insulin resistance or type 2 diabetes^3^.

The prevalence of type 2 diabetes (T2DM) has increased dramatically across the globe to 8.5% of the population in 2014, incurring tremendous human, economic and social costs. It imposes a considerable burden on society in the form of low productivity, increased healthcare expenditures, premature mortality and intangible costs in the form of a poor quality of life. The number of adults living with all types of diabetes is now over four times higher than just 40 years ago^4^. This has led the World Health Organization (WHO) to consider diabetes to be an epidemic. By 2045, the number of diabetic patients is projected to increase by 48% to over 620 million^5–7^. In 2017, the expenditures directly attributable to diabetes were approximately $727 billion, accounting for about 12% of the global healthcare expenditure on adults^5^.

The underlying reason for developing diabetes varies by type. But, regardless of type of diabetes, poor glycemic control, may, over time, lead to various potentially life threatening micro-vascular and macro-vascular complications. Approximately 40% of adults with renal disease have diabetes, while 10–15% of diabetic nephropathy patients suffer from diabetic retinopathy worldwide^8^. In addition, at least 68% of diabetic patients die from some form of cardiac disease and as many as 16% die of stroke^9^. Diabetes is therefore not only a disease in itself but is also a potentiator for many other serious conditions. In 2017, 352 million individuals were at risk of developing T2DM and 1 in 2 (212 million) individuals with T2DM went undiagnosed^5^. Worldwide, the socio-economic consequences due to the high prevalence of this disease is concerning.

Given that diabetes and its complications are preventable, the rising rate of T2DM and the complications that result from metabolic deterioration necessitate efforts to improve early detection of T2DM risk. In this context, there is a dire need for alternative approaches that: (1) are aimed at pre-emptive risk stratification and prevention, (2) provide insights needed for healthcare providers, patients, providers and health policy makers, and (3) are based on aggregated knowledge obtained from interpreting massive amount of healthcare data^10^. This need bears more weight when seeing through the fact that at least 50–80% of individuals with T2DM remain oblivious of their diagnostic status^11^. Studies reveal that 30–50% of individuals with newly diagnosed T2DM have one or more macro-vascular or micro-vascular complications at the time of diagnosis^12^.

T2DM risk prediction models along with their variants have been widely investigated^13^. In 2007, a risk scoring model was published by the Framingham offspring study to identify individuals most likely to develop T2DM in the future^14^. The Framingham Diabetes Risk Scoring Model (FDRSM) is a well known and widely used model, built using data from the Framingham heart study. The FDRSM uses point-in-time data to determine the 8-year risk for developing T2DM in an individual^15^. As such, the FDRSM allows clinicians and healthcare providers to implement intervention measures in those individuals who are at increased risk of developing T2DM. However, there are concerns when adopting risk scoring models in term of their applicability to local populations, capacity to calibrate and discriminate the model. Dekker *et al*.^16^, for instance, report, in their 2017 paper, that “*most clinical risk scores are useless*” and that “*assuming linearity of predictors*” is an example of methodological mistakes frequently made by researchers. In their 2018 paper, Steyerberg *et al*.^17^ add that these mistakes are “*quite common in current scientific practice and lead to prediction models that cannot be trusted*”.

Furthermore these scoring models are based on prospective studies (such as the Framingham heart study) that are very expensive and time consuming, especially when dealing with diseases with long latency. Therefore, we should consider and test alternative approaches to develop and validate risk models with the objective of better predicting disease risk and progression, prevent disease and allow patients to make better decisions about their health.

Machine learning (ML) techniques have shown increased relevance over the last few years and have been applied successfully to a variety of problems, including risk assessment^18,19^. ML has the potential to transform sequences of clinical measurements, as opposed to point-in-time measurements, into valuable knowledge, required for decisive steps to characterize disease risk and progression. Given that risk cumulates over time and is not a discrete state, longitudinal Electronic Medical Record (EMR) data can play a vital role in keeping track of repeated clinical measurements related to a patients’ condition over time^3^. The Hidden Markov Model (HMM) is a particularly attractive technique for assessing the temporal evolution of a disease using clinical measurements obtained from a longitudinal sample of patients in an EMR database.

We developed a HMM-based risk model that leverages longitudinal EMR data for early identification of T2DM risk in an individual. We also used the model to validate the performance of the FDRSM based on the discrimination capability of our proposed model. This could potentially result in more effective and better decision making around patient screening and proactive care with less time and investment.

## Materials and Methods

### Study design, participants and data collection

EMR data was obtained from the Canadian Primary Care Sentinel Surveillance Network (CPCSSN) which is a pioneer multi-disease EMR-based surveillance system in Canada, based at Queen’s University^20^. Data from participating networks, provided by family physicians and other primary care providers, are aggregated into a single national database (http://cpcssn.ca/). The dataset used for this study contains 812,007 records of 172,168 unique individuals, for a period ranging from August 5, 2003 to June 30, 2015, with each record containing different attributes related to demographics, diagnosis, lab results and vital signs.

All patients were assigned a reference number and were tracked for 8 years to discover their health status using this reference number only. With the exception of parental history of diabetes that were not available in our source database, the same physical and blood biochemical examinations that were addressed by the FDRSM^15^ were chosen in this study for follow up including BP (blood pressure), sex, body mass index (BMI), fasting blood glucose (FBG) levels, age, high density lipoprotein (HDL) and triglycerides (TG). Table 1 demonstrates an abstract detail of the CPCSSN dataset.

**Table 1:** Characteristics of the population in the CPCSSN database.

Predictors	Findings
**Demographic (Sex, Age)**	
Female, sample size (%)	100,566 (57)
Female age mean ± SD,Years	49.5 ± 24.8
Male age mean ± SD,Years	48.2 ± 24.1
**Vital Signs/clinical measures**	
Systolic BP, mean ± SD, mm Hg	129.34 ± 17.183
Chronic obstructive pulmonary disease, N (%)	9939 (2.4)
Dementia, N (%)	12007 (1.8)
Depression, N (%)	32672 (10)
Diabetes Mellitus, N (%)	26077 (6)
Epilepsy, N (%)	5553 (0.8)
Hypertension, N (%)	61370(13)
Osteoarthritis, N (%)	37274(7)
Parkinson’s Disease, N (%)	1825 (0.2)
**Lab Values**	
Fasting blood glucose, mean ± SD, mmol/L	5.54 ± 1.91
TG, mean ± SD, mmol/L	1.523 ± 0.962
LDL, mean ± SD, mmol/L	2.83 ± 0.99
High density lipoprotein, mean ± SD, mmol/L	1.3893 ± 0.416
BMI, mean ± SD, kg/m^2^	37.113 ± 1528.71
A1C, mean ± SD, mmol/L	6.268 ± 0.976
Cholesterol mean ± SD, mmol/L	4.893 ± 1.159

SD, Standard Deviation; Yr, Year; BP, Blood Pressure; LDL, Light Density Lipoprotein; A1C, Glycated Hemoglobin; TG, Triglycerides; BMI, Body Mass Index; HDL, High Density Lipoprotein.

*Some patients have more than 1 disease in the database.

Patients records with missing data related to any risk factors considered relevant in this study (n = 1,215) or lost to follow-up due to non-attendance at the end of the follow-up period (n = 170,042) were excluded; overall, 171,257 individuals were excluded from the research dataset. Thus, this prospective dataset resulted in a total of 911 participants ≥18 years old, of whom 61.03% were female. All of these patients had complete information related to each risk factor included in the study and did not have any differential loss to follow up. Subsequently, each record was augmented with disease status based on their health status induced from the most recent laboratory test results. Approximately 214 (23.49%) of individuals in our derived dataset were diagnosed with diabetes, of whom 52.8% were women, as depicted in Table 2.

**Table 2:** Characteristics of the derived study sample.

Predictors	Findings
**Demographic (Gender, Age)**	
Sample size without duplicates	911
Female, sample size (%)	556, (61.03)
Male age mean ± SD,Years	58.97 ± 11.96
Female age mean ± SD,Years	58.03 ± 11.02
**Vital Signs/clinical measures**	
Systolic BP, mean ± SD, mm Hg	127.611 ± 15.86
Diabetes Mellitus, N (%)	214 (23.49)
**Lab Values**	
Fasting blood glucose, mmol/L mean ± SD, mmol/L	5.573 ± 1.93
Triglycerides, mean ± SD, mmol/L	1.705 ± 1.027
HDL, sample size, mean ± SD, mmol/L	1.313 ± 0.366
BMI, mean ± SD, kg/m^2^	28.76 ± 5.818

SD, Standard Deviation; BP, Blood Pressure; BMI, Body Mass Index; HDL, High Density Lipoprotein.

All laboratory results in the CPCSSN database are recorded in mmol/L, clinical characteristics and demographics are depicted by mean ± standard deviation for categorical and continuous variables and are expressed as frequencies and percentages.

CPCSSN obtained ethics approval for all participating networks from the Health Canada Research Ethics Board and research ethics boards of all local host universities. All participating CPCSSN providers provided written informed consent for the collection and analysis of their EMR data. The PARAT tool from Privacy Analytics (Ottawa, Canada) was used to fully anonymize the data. Subsequently, Ryerson University research ethics board provided a waiver of ethics review for this study. All the methods and activities were performed in accordance with relevant guidelines and regulations.

### Proposed method

HMM is a parametric machine learning technique that has been widely deployed as a temporal latent variable model for modeling dynamic systems^21–23^. HMMs represent probability distributions over sequences of observations. Unlike Markov Chain models, none of the states are directly observable and the available data depends on hidden states via the measurement model. Before providing a probabilistic temporal prediction and evaluation of FDRSM, in accordance with our preliminary experiments, some informal insight into the structure of HMMs is given below.

Our model is assumed to be composed of the set of hidden states $S=\{{{\rm{s}}}_{1},\,{{\rm{s}}}_{2},\,{{\rm{s}}}_{3}\ldots \ldots {{\rm{s}}}_{{\rm{m}}}\}$(corresponding to diabetic or non-diabetic; usually ${{\rm{s}}}_{{\rm{i}}}$= 0 for the non- diabetic state and ${{\rm{s}}}_{{\rm{j}}}$ = 1 for the diabetic state) and a set of parameters $\theta =\{\pi ,A,\,B\}$ (explained in the paragraphs below). These parameters are then used for further analysis.

- The *prior probabilities* ${{\rm{\pi }}}_{{\rm{i}}}=\{{{\rm{q}}}_{1}={{\rm{s}}}_{{\rm{i}}}\}\,\,$are the probabilities of ${{\rm{s}}}_{{\rm{i}}}$ being the first state of a system or seeing the first real state ${{\rm{s}}}_{{\rm{i}}}$ as P(${{\rm{s}}}_{{\rm{i}}}$| ${{\rm{s}}}_{0}$). Collected in a vector ${\rm{\pi }}$ and the ${{\rm{s}}}_{{\rm{i}}}$ coordinates of ${\rm{\pi }}$ should be interpreted as the initial state of the system.
- The *transition probabilities matrix (A)* are the probabilities to go from state $$i$$ to state $$j$$ at time $$t$$: $${{\rm{a}}}_{{\rm{i}},{\rm{j}}}=$$$$p({{\rm{q}}}_{{\rm{t}}+1}={{\rm{s}}}_{{\rm{j}}}|{{\rm{q}}}_{{\rm{t}}}={{\rm{s}}}_{{\rm{i}}})$$. The prognosis or the course of a disease can be specified by the transition matrix A. The transition matrix consists of $${{\rm{a}}}_{{\rm{i}},{\rm{j}}}$$ that denote the conditional probability or the rate about the system transitions from $${{\rm{s}}}_{{\rm{i}}}$$ to $${{\rm{s}}}_{{\rm{j}}}$$, whereas the probability of $${{\rm{s}}}_{{\rm{j}}}$$ at time $$t$$ depends solely upon on $${{\rm{s}}}_{{\rm{i}}}$$ at time $$\,{\rm{t}}-1$$. In our proposed system transition probabilities consist of a square matrix of order m = 2 and must hold the following properties^24^.

1 $$0\le {{\rm{a}}}_{{\rm{i}},{\rm{j}}}\le 1\,,{\rm{i}},\,{\rm{j}}=1,2,3\ldots \ldots .{\rm{m}}$$

2 $$\,{{\sum }^{}}_{{\rm{j}}=1}^{{\rm{m}}}{{\rm{a}}}_{{\rm{i}},{\rm{j}}}=1,\,{\rm{i}}=1,2,3,\,\ldots \mathrm{..}{\rm{m}}$$

- The *emission probabilities matrix (B)* characterize the likelihood of a certain observation ${{\rm{o}}}_{{\rm{i}}}$, if the model is in state ${{\rm{s}}}_{{\rm{i}}}$. The HMM we use involves observed variables ${{\rm{V}}}_{k}\,{\rm{and}}\,{\rm{K}}=1,2,\ldots .{\rm{n}}$,whereas n = 6, that are conditioned upon the hidden states at time *t*. The choice of observed variables used in this study is based on the FDRSM. The fundamental principle behind HMM is the estimation of an optimal hidden state sequence of a process using observed variables over time, whereas the observed variables have no one-to-one relationship with hidden states but are associated through the probability distribution.

As the data set used in this study contained continuous valued observations

$O=\{{O}_{t}^{(l)},\,t=1,2,,\ldots .T,\,l=1,2,3,\,\ldots .L\}$ and ${{\rm{O}}}_{{\rm{t}}}\epsilon {{\rm{R}}}^{{\rm{D}}}$ where *T* is the length of each sequence and *l* is the numbers of independent observation sequences. We retained risk factors values as continuous, as transforming continuous variables into discrete categories by putting them in class intervals resulted in loss of information in discovering the true underlying association among latent states and observable factors^25^. Thus, the observation probability assumes the Gaussian distribution, then we have a continuous HMM with ${{\rm{b}}}_{{\rm{i}}}({\rm{K}})={{\rm{b}}}_{{\rm{i}}}({{\rm{O}}}_{{\rm{t}}}={{\rm{V}}}_{{\rm{k}}})={\mathscr{N}}({{\rm{V}}}_{{\rm{k}}},\,{\mu }_{{\rm{i}}},\,{{\rm{\sigma }}}_{{\rm{i}}}),$ where ${\mu }_{{\rm{i}}}$ and ${{\rm{\sigma }}}_{{\rm{i}}}$ are the mean and variance of the distribution corresponding to the state ${{\rm{s}}}_{{\rm{i}}}$, respectively, and ${\mathscr{N}}$ is the probability density function that can be defined as follows:

3 $$p(x|\mu ,\,{\rm{\sigma }})={\mathscr{N}}\,(x|\mu ,\,{\rm{\sigma }})={\frac{1}{\sqrt{2{\rm{\pi }}{\rm{\sigma }}}}}_{0}\exp (\frac{-{(x-\mu )}^{2}}{2{\rm{\sigma }}})$$

Then, HMM is specified by

4 $${\rm{\lambda }}=\{{\rm{A}},\,\mu ,\,{\rm{\sigma }},\,\pi \}$$

**Figure 1:**
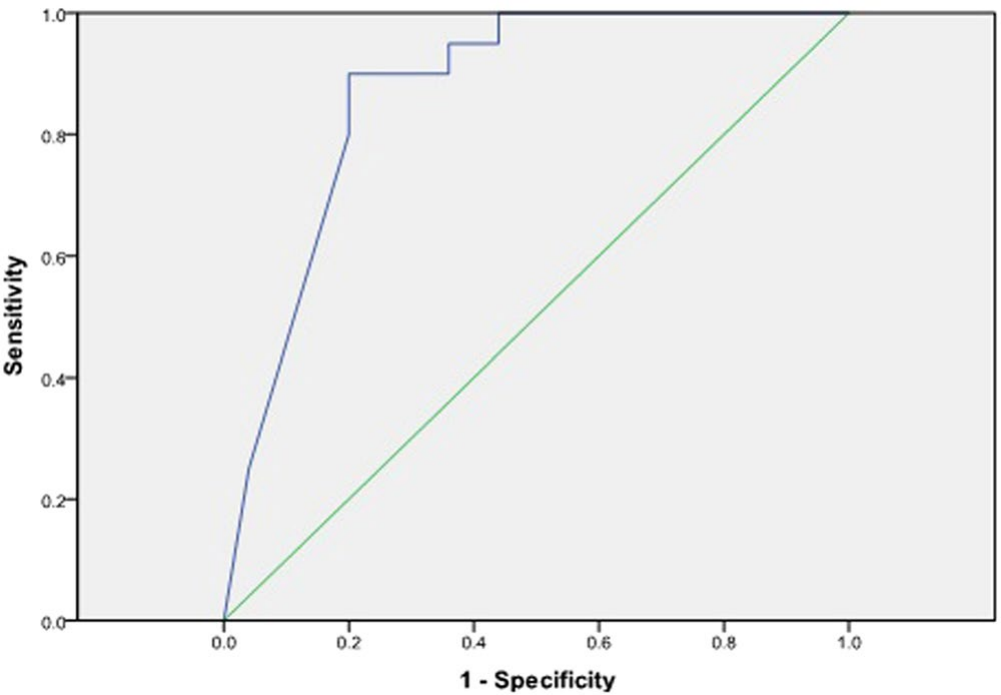
The receiver operating characteristic curve (AROC) of our proposed model over derived study sample.

The vectors of μ and σ for the proposed system along with the initial probability and transition probability matrixes are provided in Supplementary File 1. All experiments and statistical analyses were performed using IBM SPSS Statistics (version 19) and Python (Version 2.7). Once the dataset was prepared and the parameters drawn from the training set, the viterbi algorithm from the Hidden Markov Model API (Hmmlearrn) was used to train the GausianHMM to evaluate the 8-year risk of developing T2DM in an individual. Several variants of the basic HMM have been proposed, with slightly different functionality. The basic concept was published in a series of classic papers by Baum *et al*.^26^. The area under the receiver operating characteristic curve (AROC) is used to determine the effectiveness of our proposed approach. Figure 1 demonstrates the result of the AROC in our derived study sample.

## Results

We also performed multiple regression analysis to find the significant p-value for individual risk factors for developing T2DM. According to this statistical analysis, all the risk factors were statistically significant (Nagelkerke *R*^2^*(7)* = 0.546). However, the association of gender and T2DM was not overly strong, with an odds ratio of 0.552 as depicted in Table 3. The Framingham study excluded age and gender variables from the diabetes risk calculation because of their negative significance. However, we only exclude gender from risk factors and calculate the overall 8-year risk for developing T2DM including blood pressure, fasting blood glucose, triglycerides, HDL, BMI and age.

**Table 3:** Association between individual risk factors and T2DM in the derived dataset.

Explanatory variables	OR (95% C.I.)	*P* Value
Age	1.006 (0.993–1.020)	0.000
Male	0.552 (0.472–0.701)	0.030
Systolic blood pressure	0.998 (0.988–1.008)	0.00
BMI	1.011 (0.985–1.038)	0.022
HDL	0.601 (0.312–0.803)	0.004
Triglycerides	1.076 (0.862–1.343)	0.002
Fasting blood glucose	9.936 (7.638–12.925)	0.000
Intercept		0.000

Nagelkerke R^2^ = 0.546.

Hosmer and Lemeshow Test = 0.360 (Significantly greater than 0.0005).

OR, Odds Ratio; C.I. confidence Interval; BMI, Body Mass Index; HDL, High Density Lipoprotein.

Table 4 demonstrates a comparison among our final derived dataset, the Framingham research study sample^14^ and the validation study of the FDRSM in the Canadian population^15^. The average age of our study sample is 58.97 years and the overall BMI average is 28.76. The cases with systolic blood pressure >130/80 mm Hg, number of women, average age and BMI, as well as impaired glucose tolerance, are greater than those of the Framingham research sample. Nevertheless, the number of cases with triglycerides levels greater than or equal to the cutoff point (Triglycerides levels ≥1.7 mmol/L) are also high but cases with HDL levels <0.9 mmol/L in male and <1.2 mmol/L in female is much lower than that of the Framingham research sample.

**Table 4:** Comparative analysis of our derived research sample with the Framingham study and validation study of FDRSM in Canadian population research samples.

	Research sample in our study	Framingham simple clinical model	Research sample of validation study of FDRSM in Canadian population
Sample size	911	3140	1970
Female (%)	61.03	53.9	60.6%
Age mean, SD,Years	58.97 ± 11.965	54.0 ± 9.8	56.60(5.29)
Systolic BP >130/85 mm Hg,%	49	44.2	20.1
Triglycerides levels ≥1.7 mmol/L,%	53	31.8	27.9
HDL levels <0.9 mmol/L in male and <1.2 mmol/L in female,%	17	36.9	18.9
Fasting blood glucose levels 5.5 to 6.9 mmol/L, %	47	27.0	30.3
BMI, mean ± SD, kg/m^2^	28.76 ± 5.818	27.1 ± 4.7	28.28(6.07)

SD, Standard Deviation; BP, Blood Pressure; BMI, body mass index; HDL, high-density lipoprotein.

We utilized the jackknife or “leave one out” procedure in order to build HMM. It is a cross-validation technique first developed by Quenouille^27^, widely used to evaluate the actual predictive power of computational predictive model and to minimize the risk of over-fitting. Technically, the goal is to estimate the generalization performance of a predictive model as a random effect model. This is done by dropping in turn each observation and fitting the model for the remaining set of observations. The model is then used to predict the left-out observation. With this procedure, each observation has been predicted as a new observation. Gong^28^ provided a detailed description of the jackknife procedure. Following the jackknife procedure, we randomly selected 3 different validation partitions with 45 samples of 90% of the participants to evaluate the discriminability of the proposed model in order to estimate the 8-year risk of developed T2DM. Subsequently, to evaluate the predictive performance of the multivariate HMM, we used AROC as a similarity measure. The AROCs for these validation datasets ranged from 0.73 to 0.869, demonstrating a high reliability of discrimination for the HMM model in repeated random-sample subsets. However, the final model was chosen based on the performance, both in terms of standard error and general behavior in each patient, on the validation dataset.

Figure 1 shows the predictive power of our proposed model over derived study sample, as described above, in term of AROC. Theoretically, the AROC can assume values between 0 and 1. However, the practical lower bound for a random classifier is 0.5, implying no discriminative capability, while an ideal classifier will take the value of 1. Figure 1 demonstrates graphically that we have a curve that mimics the Bell curve which means we have a large area under the curve. It has a good balance of sensitivity and specificity with an AROC value of 0.869 which is statistically significant with p-value < 0.05 and a narrow 95% Confidence Interval (CI).

**Figure 2:**
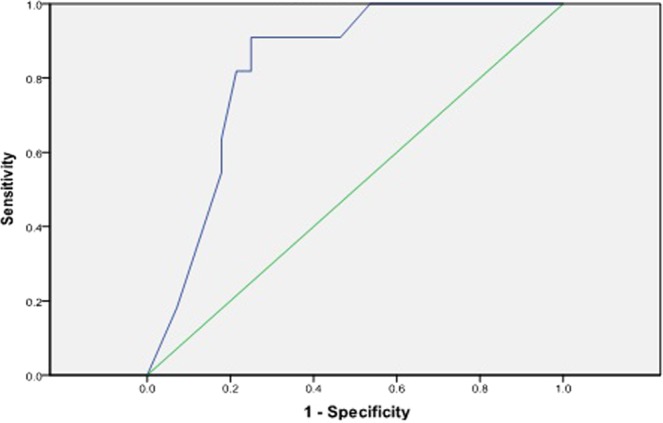
The receiver operating characteristic curve (AROC) of our proposed model over derived study sample excluding age as one of the contributing risk factor.

The AROC in our research sample, using the proposed approach, was 86.9% (p < 0.0005, Standard Error = 0.54 [95% CI, (0.763–0.975)]), as shown in Table 5. The proposed method was also evaluated and compared to baseline approaches as depicted in Table 6. It demonstrated a comparative analysis of the AROCs and the risk for developing diabetes within 8 years among our research sample, the Framingham study research sample using their simple clinical model^14^ and the validation study of the Framingham risk scoring model on a Canadian population^15^ with AROCs of 86.9%, 85% and 78.6% respectively. Furthermore, experimental results demonstrates that the AROC of our proposed model is superior to the model developed for the FDRSM validation study in a Canadian population^15^ and the Framingham simple clinical model^14^. It can also be concluded that machine learning techniques have the potential to validate complex models based on prospective studies with high performance and are capable of identifying persons who will develop T2DM from those who will not. Whereas Fig. 2 represents AROC (p < 0.0005, Standard Error = 0.60 [95% CI, (0.710–0.946)]) of our proposed model using risk factor consider relevant in this study excluding age, to determine the significance of age in developing diabetes risk.

**Table 5:** Summary of Area Under Receiver Operating Characteristic Curve (AROC) in our derived research dataset.

	AROC	Std. Error^a^	Asymptotic Sig.^b^	Asymptotic 95% Confidence Interval	
				Lower Bound	Upper Bound
Over the derived study dataset	0.869	0.054	0.000	0.763	0.975
Over the derived dataset, excluding age	0.828	0.60	0.000	0.710	0.946

The test result variable(s): cal has at least one tie between the positive actual state group and the negative actual state group. Statistics may be biased.

Under the non parametric assumption.

Null hypothesis: true area = 0.5.

**Table 6:** The comparative analysis of AROCs and 8-year risk for developing diabetes among our research sample, the Framingham research sample (simple clinical model) and FDRSM validation study in Canadian population.

	Proposed HMM based risk model	Framingham simple Clinical model	Validation study of FDRSM in Canadian population
AROC, %	86.9	85.0	78.6
<3, %	42.2	63.8	70.1
3 to 10, %	44.4 (between 3 to 9)	20.7	16.3
>10, %	13.3 (equal to 10)	15.6	13.6

AROC; Area Under receiver Operating Characteristic Curve.

According to the probabilistic prediction of HMM, we determined that 42% of individuals in our sample had a risk of less than 3%; 44% had a risk between 3% and 9% and 13% had a risk for developing diabetes within 8 years greater than or equal to 10%. The estimated risk figures, amongst the 3 different risk categories with the cutoff value < 3, 3 to 10 and greater than 10, for developing type 2 diabetes within 8 years in the Canadian validation study of the FDRSM were respectively 70.1, 16.3 and 13.6, whereas the Framingham study predicted 63.8%, 20.7% and 15.6% respectively, as depicted in Table 6.

As the performance of our proposed model was comparatively good, we also estimated the 8-year risk of T2DM for 1458 non-diabetic individuals for whom data was available in 2015. We determined that in our baseline dataset we can identity at least 16.9% (247) individuals at increased high risk for developing T2DM in the 8-year interval ranging from 2015 to 2022.

## Discussion

The increase in T2DM incidence is the main reason for increased diabetes prevalence around the world. It has a prolonged latent phase particularly in its early period and is thus poorly controlled^29^. Several meta analyses and clinical trials convincingly suggest that early interventions can postpone or prevent T2DM^30,31^. Early identification of high risk patients even when they are in a normoglycemic state is highly desirable, since interventions to prevent diabetes take time to implement. From a clinician and payor prospective, the development of such risk assessment techniques could enable optimal allocation of resources and healthcare services with greater confidence^32^. Although traditional risk factors for diabetes offer general guidance, they are ineffective for individual risk assessment^33^.

Several risk scoring models have been widely investigated to identify patients at high risk for developing T2DM as well as to communicate risk estimated effectively. Among them, the FDRSM is a well-known and widely used diabetes risk scoring model. This model was proposed to predict the 8-year risk of developing diabetes risk in middle-aged adults using 6 risk factors, including BMI, FBG, positive parental history of diabetes HDL, blood pressure and TG^14^. The FDRSM is primarily based on the data obtained from Framingham heart study. Technical details about the FDRSM and interactive risk calculator can be found on the Framingham heart study website (https://www.framinghamheartstudy.org).

The Framingham Heart Study is the first, most comprehensively characterized multigenerational and ongoing study of its kind. It continues to provide an effective platform for the primary prevention of chronic diseases. It has contributed to a paradigm shift in the history of medicine through its community-based approach. Despite of major contributions, this observational study is consuming a lot of resources and time. In such scenarios, special-purpose techniques are required. In line with the suggestion of the original paper, the Framingham offspring study, the FDRSM risk scoring model should be tested in various populations in order to ensure its validity in local population, Mashayekhi *et al*.^15^ proposed a study to validate the performance of the FDRSM in a Canadian population. The reported AROC was 78.6%, which is fair, given that parental history of diabetes was omitted because it was not available in the CPCSSN database. However, in the present study an effort has been made (1) to develop a HMM based diagnostic predictive model for leveraging EMRs data by utilizing temporal evolution of diabetes progression captured in repeated clinical measurements obtained from a longitudinal sample of patients (2) to validate the performance of the FDRSM as well as avoid some of the above mentioned limitations in order to assist health care professionals/physicians in investigating the 8-year risk of developing T2DM in an individual with the objective to control and manage the downstream consequences of diabetes. Unlike traditional machine learning techniques, the proposed HMM model has the ability to provide explicit information about prognosis, while utilizing the inherent temporal dependencies present in the data, and which is required to characterize disease risk and progression over time.

Our comparative analysis using a dataset with and without age, demonstrates that age does exhibit a significant association with diabetes risk, as slight under performance does occur when age is excluded from the dataset.

Unfortunately, this finding does not provide much guidance for T2DM prevention as age, along with sex, are non-modifiable risk factors. The remaining risk factors included in risk stratification are meaningful for the implementation of preventive and interventional measures in order to decrease the incidence of diabetes. Existing literature also highlights that modifiable risk factors contribute significantly to reduced risk of developing T2DM^34^. The Framingham study determined odds ratios of 1.00 and 1.15 for triglycerides and fasting blood sugar for predicting the 8-year risk of developing T2DM. The results of our study are consistent with the results of the Framingham study with respect to triglycerides (odds ratio 1.076 [95% CI, 0.862–1.343], p < 0.005). However, in our study sample fasting blood sugar demonstrated an overly strong association with diabetes onset (9.936 [95% CI, 7.638–12.925], p < 0.005). All other risk factors included in this study were also significantly associated with the incidence of diabetes. Comparative analysis of the percentage of people with low HDL levels in the Framingham research sample in Table 4 implies that the cut-off values for HDL should be revisited.

Validation of a risk-score model often involves plotting observed cases verses estimated probability^35^. We found an overlap between observed incidence and estimated probability in our analysis. Thus, estimated risk has a certain accuracy, however discrimination is the ability of the model to differentiate between individuals who have the disease from those who do not. We included the AROC analysis to evaluate the discriminatory capability of our proposed model to identify the 8-year risk of developing T2DM. The reported AROC for the proposed study is 86.9%, which is comparatively good, given that diabetic parental history is omitted due to its unavailability in the dataset. Experimental results also demonstrated that our proposed model has the potential to effectively predict the 8-year risk of developing T2DM in an individual.

These results are significant because in addition to identifying a-priori T2DM risk, this is the first study to evaluate the performance of the Framingham diabetes risk scoring model using a state of the art HMM. We believe this will motivate future investigations to apply ML methods to EMR data to assist in identifying the risk of developing various other diseases. The proposed method can be used easily by healthcare providers to identify high risk patients who may benefit from intensified prevention and intervention measures and as a result, halt or delay the onset of diabetes with reduced healthcare expenditure and improved healthcare services delivery.

It is estimated that people with diabetes are 2.6 times more likely to be hospitalized in the past year than people without diabetes (21% vs. 8%)^36,37^. The approximate healthcare expenditure for an individual with diabetes in the US is ∼$16,750 per year, of which ∼$9,600 is the direct cost of diabetes^38^. Economic costs and social burden of diabetes estimated by the American Diabetes Association demonstrates that the costs of diabetes increased by approximately 200% from 2002 to 2012^38^. Given the newly predicted high risk individuals, a substantial fraction of healthcare cost and individual disease burden in our baseline dataset could be saved if clinicians and healthcare providers manage those high risk individuals promptly.

Despite the promising results, our study has several limitations. First, parental history of diabetes is missing in our model. This affects the internal validity of our proposed model. In addition, as our study sample only contains information related to those risk factors that were addressed by the FDRSM^14,39,40^, other risk factors incorporated in various risk scoring models are ignored (like, diet, physical activity, smoking, alcohol consumption and ethnicity). Second, the dataset used in this research is mainly obtained from a Canadian population; caution is required when generalizing these findings to other populations.

## Conclusion

T2DM imposes inexorable and significant burdens on society in term of lost productivity, premature mortality, and intangible costs in the form of poor quality of life. Risk stratification is central to identifying and managing individuals at increased risk for developing diabetes. The major contribution of this research consists in developing an HMM to extract predictive information from temporal sequences of clinical measurement in order to determine a-prior 8-year risk of developing T2DM in comparison to the standard FDRSM. Compared to an established risk scoring model, the results of this study demonstrated that HMM, a machine learning technique, significantly improves the accuracy of T2DM risk prediction by exploiting complex interactions between risk factors. The proposed technique has the potential to be used in healthcare settings to identify potentially vulnerable individuals who could most likely benefit from preventive treatment, while avoiding unnecessary treatment for those who are at low risk.

## Supplementary information

Supplementary file
