# Supplementary material for: Prognostic Modeling and Prevention of Diabetes Using Machine Learning Technique
Source: Sci Rep. 2019 Sep 24;9:13805. doi: 10.1038/s41598-019-49563-6 (PMC6760163; doi:10.1038/s41598-019-49563-6)
Supplement: Supplementary file 1 — Supplementary file [file 41598_2019_49563_MOESM1_ESM.docx]

**Prognostic Modeling and Prevention of Diabetes Using Machine Learning Technique**

**Sajida Perveen^a^, Muhammad Shahbaz^a,b^, Karim Keshavjee^b,c^, Aziz Guergachi^b,d,e^**

^a^Department of Computer Science & Engineering, University of Engineering & Technology, Lahore, Pakistan

^b^Research Lab for Advanced System Modelling, Ryerson University

^c^Dalla Lana School of Public Health, University of Toronto, Toronto, Ontario, Canada

^d^Ted Rogers School of Information Technology Management, Ryerson University, Toronto, Ontario, Canada

^e^Department of Mathematics & Statistics, York University, Toronto, Ontario, Canada

We initialize the HMM parameters according to the EMRs data obtained from CPCSSN database. The input data stream is sorted by time and feed into the algorithm to calculate initial probability matrix, transition probability matrix and vectors of mean and variance.

We simply count the records of each type to derive initial probabilities of each latent state. The obvious alternative is to treat all state transitions and each element in as equally probably (0.5 each on a two-state model). We consider our initial values, derived from the data, to be more useful in that they are bound to our understanding of the observed events in our data; that is, we consider them as actual pairs of events and, from this empirical understanding, infer their most appropriate hidden state context.

$$S=\left[ \begin{matrix} s_{\mathrm{diabetic}} \\ s_{\mathrm{non}-\mathrm{diabetic}} \end{matrix} \right]$$

The transition matrix consists of $a_{i,j}$ that denote the conditional probability or the rate about the system transitions from $s_{i}$ to $s_{j}$, whereas the probability of $s_{j}$ at time $t$ depends solely upon on $s_{i}$ at time$t-1$. In our proposed system transition probabilities consist of a square matrix of order m = 2

$$A=\left[ \begin{matrix} a_{\mathrm{non}-diabetic, non-\mathrm{diabetic}} & a_{\mathrm{non}-diabetic, diabetic} \\ a_{diabetic, non-\mathrm{diabetic}} & a_{diabetic, diabetic} \end{matrix} \right]$$

As the data set used in this study contained continuous valued observations. We retained risk factors values as continuous, as transforming continuous variables into discrete categories by putting them in class intervals resulted in loss of information to discover true underlying association, further obtaining optimistic results. Thus, the observation probability assumes the Gaussian distribution.

Then we have a continuous HMM with $b_{i}\left( K \right)= b_{i}\left( O_{t}=V_{k} \right) =\mathcal{N}(V_{k}, \mu_{i}, \sigma_{i}),$ where $\mu_{i}$ and $\sigma_{i}$ are the mean and variance of the distribution corresponding to the state $s_{i}$, respectively, and $\mathcal{N}$ is probability density function.

The set of hidden corresponding to diabetic or non-diabetic; $s_{i}$= 0 for the non- diabetic state and $s_{j}$ = 1 for the diabetic state. Whereas risk factors are as follow:

Age, systolic blood pressure, body mass index, high density lipoprotein, triglycerides and fasting blood glucose respectively.

Initial probability: {0: 0.7, 1: 0.3}

Transition probability: {0: [0.8, 0.2], 1:[0.1, 0.9]}

Mean vector against each risk factor by class value: {0.0: [58.1259622113366, 126.55773268019594, 28.26136639102868, 1.4833589920083972, 1.2923652906508016, 5.190552834149755]; {1.0: [(61.8177570093458, 129.96495327102804, 30.462130748014026), 1.2634112156542048, 1.6534579439485988, 6.847897196261677]}

Variance vector against each risk factor by class value: {0:[12.003656270775107, 15.741844339307924, 5.85640404765087, 0.42283993614520277, 0.6609985259212197, 0.5093814995536743], 1:[ 11.39474642153458, 15.737935366485804 , 5.355720864928974, 0.3182314265047891, 0.7653192405320948, 1.7894879800167522]}
